# Effect of *CHRFAM7A Δ2bp* gene variant on secondary inflammation after spinal cord injury

**DOI:** 10.1371/journal.pone.0251110

**Published:** 2021-05-06

**Authors:** Mingkuan Lin, Wan Huang, Nadine Kabbani, Mark M. Theiss, John F. Hamilton, James M. Ecklund, Yvette P. Conley, Yoram Vodovotz, David Brienza, Amy K. Wagner, Emily Robbins, Gwendolyn A. Sowa, Robert H. Lipsky

**Affiliations:** 1 School of Systems Biology, George Mason University, Fairfax, Virginia, United States of America; 2 Inova Neuroscience and Spine Institute, Inova Health System, Falls Church, Virginia, United States of America; 3 Department of Physical Medicine and Rehabilitation, University of Pittsburgh School of Medicine, Pittsburgh, Pennsylvania, United States of America; 4 Department of Orthopedic Services, Inova Health System, Falls Church, Virginia, United States of America; 5 School of Nursing and Department of Human Genetics, University of Pittsburgh, Pittsburgh, Pennsylvania, United States of America; 6 Department of Surgery, Center for Inflammation & Regenerative Modeling in McGowan Institute for Regenerative Medicine, University of Pittsburgh, Pittsburgh, Pennsylvania, United States of America; 7 Rehabilitation Science &Technology, Bioengineering, McGowan Institute for Regenerative Medicine, University of Pittsburgh, Pittsburgh, Pennsylvania, United States of America; University of Louisville, UNITED STATES

## Abstract

The α7 neuronal nicotinic acetylcholine receptors (α7nAChRs) are essential for anti-inflammatory responses. The human-specific *CHRFAM7A* gene and its 2bp deletion polymorphism (*Δ2bp* variant) encodes a structurally-deficient α7nAChRs that may impact the anti-inflammatory function. We studied 45 spinal cord injury (SCI) patients for up to six weeks post SCI to investigate the role of the *Δ2bp* variant on multiple circulating inflammatory mediators and two outcome measures (neuropathic pain and risk of pressure ulcers). The patient’s SCI were classified as either severe or mild. Missing values were imputed. Overall genetic effect was conducted with independent sample t-test and corrected with false discovery rate (FDR). Univariate analysis and regression analysis were applied to evaluate the *Δ2bp* effects on temporal variation of inflammatory mediators post SCI and their interaction with outcome measures. In severe SCI, the *Δ2bp* carriers showed higher levels of circulating inflammatory mediators than the *Δ2bp* non-carriers in TNF-α (FDR = 9.6x10^-4^), IFN-γ (FDR = 1.3x10^-3^), IL-13 (FDR = 1.6x10^-3^), CCL11 (FDR = 2.1x10^-3^), IL-12p70 (FDR = 2.2x10^-3^), IL-8 (FDR = 2.2x10^-3^), CXCL10 (FDR = 3.1x10^-3^), CCL4 (FDR = 5.7x10^-3^), IL-12p40 (FDR = 7.1x10^-3^), IL-1b (FDR = 0.014), IL-15 (FDR = 0.024), and IL-2 (FDR = 0.037). IL-8 and CCL2 were negatively associated with days post injury (DPI) for the *Δ2bp* carriers (P = 2x10^-7^ and P = 2x10^-8^, respectively) and IL-5 was positively associated with DPI for the *Δ2bp* non-carriers (P = 0.015). Neuropathic pain was marginally positively associated with IL-13 for the *Δ2bp* carriers (P = 0.056). In mild SCI, the *Δ2bp* carriers had lower circulating levels of IL-15 (FDR = 0.04) than the *Δ2bp* non-carriers. Temporal variation of inflammatory mediators post SCI was not associated with the *Δ2bp* variant. For the mild SCI *Δ2bp* carriers, risk of pressure ulcers was positively associated with circulating levels of IFN-γ, CXCL10, and CCL4 and negatively associated with circulating levels of IL-12p70. These findings support an important role for the human-specific *CHRFAM7A Δ2bp* gene variant in modifying anti-inflammatory function of α7nAChRs following SCI.

## Introduction

Individuals with traumatic spinal cord injury (SCI) are subjected to severe motor and/or sensory dysfunction and have a poorer quality of life outcome. SCI results in neurological deficits through both primary and secondary damage. Primary damage is caused by the direct mechanical impact to spinal cord tissue immediately after injury. Secondary damage is driven by the inflammation incurred after the primary injury and continues for several days or months. Primary damage is usually irreversible and therefore the main therapy is focused on controlling inflammation to prevent further damage to tissues due to inflammation. Cytokines released from resident CNS microglia and by infiltrating immune cells, namely neutrophils and monocytes/macrophages. In addition, neutrophils and macrophages release proteases, including matrix metalloprotease 9 (MMP-9), which alters the blood-spinal cord barrier and induces neuronal axon dieback, contributing to further injury. These cells also release reactive oxygen intermediates, lysosomal enzymes, and pro-inflammatory cytokines/chemokines to aggravate cellular damage [1, 2]. The complex role of inflammation after injury, providing certain beneficial aspects initially, but also causing damages to surrounding tissue, inducing apoptotic cell death, and impairing spontaneous neuron regeneration and functional recovery [3, 4].

The cellular responses to the primary mechanical injury produce a secondary cascade of cellular and biochemical events that activate resident astrocytes and pericytes, and recruitment of fibroblasts and Schwann cells from the periphery, leading to the development of lasting glial and fibrotic scars in the injured spinal cord and the development of a lasting pro-inflammatory state [5, 6]. Partial or complete interruption of communication from the brain to levels below the injury results in a disruption of descending motor tracts, resulting in paralysis. Other common features of SCI, such as pain, spasticity, autonomic dysreflexia, loss of bladder and bowel control, as well as respiratory and sexual function add to impaired quality of life. Repairing or restoring function is of high priority. Presentation of severe SCI pain, which develops in approximately two-thirds of SCI patients and is classified as acute if it is <12 weeks, and chronic if it is >12 weeks and persists despite an apparent lack of ongoing injury [7]. Chronic pain is another source of poor quality of life because it is frequently non-responsive to current treatments. SCI pain is also characterized as being either nociceptive, as a result of pain from nociceptors, or NP, which is the result of somatosensory nerve damage. As a consequence of variability of SCI outcomes, the use of protein and genetic biomarkers associated with SCI pathology or recovery may offer greater predictive value for identifying SCI patients at risk for developing NP.

The vagus nerve is generally recognized in regulating inflammation after injury by activating α7 neuronal nicotinic acetylcholine receptors (α7nAChRs, encoded by the *CHRNA7* gene) ion channel on leukocytes and other immune cells. Functional α7nAChRs expression enables systemic and local anti-inflammatory responses to an injury [8–10]. Knockout of an ortholog of the *CHRNA7* gene in mice renders vagus nerve stimulation ineffective after injury, suggesting that expression of the *CHRNA7* gene is essential for anti-inflammatory responses [11]. In peripheral systems, activation of α7nAChRs acts as the modulator of innate inflammatory responses to inhibit the release of pro-inflammatory cytokines [12, 13]. In addition to anti-inflammatory effects, activation of α7nAChRs reduces NP in a rat model of chronic pain via microglia [14].

The human genome uniquely has a *CHRFAM7A* gene that shares exons of the *CHRNA7* gene [15, 16] and had shown close association of *CHRFAM7A* and *CHRNA7* expression in human leukocyte and specific inflammation response [17]. Structurally, the *CHRFAM7A* gene shares exons 5–10 of the *CHRNA7* gene and has a reverse transcriptional orientation with respect to the *CHRNA7* gene. Most of the population (77%) maintains two copies of both *CHRNA7* and *CHRFAM7A*, while approximately 19% of the population maintains two copies of *CHRNA7* and one copy of *CHRFAM7A*, and approximately 7% of the population carries 2 copies of *CHRNA7* but none of *CHRFAM7A* [18–20]. A small 2bp deletion in exon 6 of *CHRFAM7A* (*CHRFAM7AΔ2bp*, rs67158670) [15], which is not present in *CHRNA7*, is associated with a gene inversion and has the same orientation as the parent *CHRNA7* gene [20, 21]. The *CHRFAM7AΔ2bp* has a population distribution of approximately 42% in Caucasians and 14% in African Americans [20].

The α7nAChRs are homopentameric cation conduction channels structurally related to a family of cysteine bridge (cys-loop) containing receptors [22]. The α7nAChR ion channel subunit encoded by *CHRNA7* gene has three glycosylation sites and a cysteine bridge. However, the α7 polypeptide subunit encoded by *CHRFAM7A* gene would be missing one glycosylation site and the α7 polypeptide encoded by *CHRFAM7AΔ2bp* lacks all of the glycosylation sites as well as the cysteine bridge [20]. Expression of *CHRNA7* and *CHRFAM7A* encoded subunits in a 1:1 molar ratio in *Xenopus* oocytes resulted in a 30% decrease in acetylcholine-stimulated current [23, 24], suggesting that the *CHRFAM7A* acts as a dominant-negative regulator of ion channel function. The *CHRFAM7AΔ2bp* encoded polypeptide is an even more potent inhibitor of α7nAChR function than the wild-type *CHRFAM7A* encoded subunit in its inability to flux Ca^2+^ [23]. Thus, the *CHRFAM7AΔ2bp* carrier status of individuals with *CHRFAM7A* may predict a dominant modulator to weaken the anti-inflammatory role of α7nAChRs in the human innate immune response. In a recently published study from our research group in a small cohort of SCI subjects with severe injury and emergency room consented controls, we found that TNFα levels, as a representative cytokine of elevated inflammatory mediators, were nearly three-fold higher in SCI subjects carrying the del2bp variant of the *CHRFAM7A* gene compared to SCI subjects having the no deletion genotype (p = 0.001 ANOVA) as determined three weeks post-injury [25]. Individuals with the del2bp variant also had significantly increased levels of NP. Taken together, these data support the idea that genetic variation of *CHRFAM7A* gene may affect clinical outcomes after SCI.

The present study we expanded our previous work to investigate the role of the *CHRFAM7AΔ2bp* variant in the inflammatory in SCI subjects stratified by injury severity. We hypothesized that following SCI, people with the *CHRFAM7AΔ*2bp variant would sustain a greater pro-inflammatory response relative to individuals who do not carry this variant. We prospectively collected biochemical data for a spectrum of plasma inflammatory mediators from SCI subjects over time after injury. We also obtained *CHAMRF7AΔ2bp* genotypes in the same groups of subjects. We then evaluated the potential influence of SCI severity, genetics, temporal effects in inflammatory responses after SCI and clinical outcomes. For reference, the *CHRFAM7AΔ2bp* polymorphism (rs67158670) is referred to as the *Δ2bp* variant in subsequent paragraphs.

## Materials and methods

### Participants

A total of 45 SCI patients (seven females and thirty-eight males, age 18–67 years) were included in this study and recruited from November 2009 to October 2011 at the University of Pittsburgh. The study was approved by the Institutional Review Boards of the University of Pittsburgh and the Inova Health System. The cohort of 45 traumatic SCI subjects with severity defined by American Spinal Injury Association (ASIA) Impairment Scale (AIS) [26]. AIS grade A and B injuries were grouped as severe SCI (25 patients) and AIS grade C and D injuries were grouped as mild SCI (20 patients). Patients with MRI-confirmed diagnosis of acute traumatic SCI with written informed consents were included. The exclusion criteria were pre-existing immune system related diseases and medications (including anti-inflammation or immune suppression treatments) as well as any previous SCI or other neurological diseases that affect motor and sensory function, or non-traumatic etiology. All medications taken during enrolment were recorded. This study was carried out in accordance with the recommendations of the Institutional Review Boards of the University of Pittsburgh and INOVA Health System with written informed consent from all subjects. All subjects gave written informed consent in accordance with the Declaration of Helsinki. The protocol was approved by the Institutional Review Boards of the University of Pittsburgh and INOVA Health System.

### General management

Patients were treated in accordance with guidelines for the management of SCI, including intensive care unit (ICU) monitoring, tests for sensory function and movement, early (< 48 hour) intervention for SCI treatment, and surveillance for pneumonia.

### Biochemical analyses

Patient blood samples were obtained 1–3 times per week post injury (first blood draw 4.66±3.77 days). Blood samples were collected into EDTA coated tubes via central venous catheters and centrifuged to obtain plasma for biochemical analyses. The human inflammatory MILLIPLEX ™ MAP Human Cytokine/Chemokine Panel-Premixed 23 Plex (Millipore Corporation, MA, USA) and Luminex™ 100 IS (Luminex, TX, USA) was used to measure plasma levels of interleukin IL-1β, IL-1 receptor antagonist (IL-1ra), IL-2, soluble IL-2 receptor-α (sIL-2Rα), IL-4, IL-5, IL-6, IL-7, IL-8 (CCL8), IL-10, IL-12 (IL-12p70), IL-12p40, IL-13, IL-15, IL-17, interferon IFN-γ, IFN-γ inducible protein (IP-10; CXCL10), monokine induced by gamma interferon (MIG; CXCL9), macrophage inflammatory protein (MIP-1α; CCL3 and MIP-1β;CCL4), monocyte chemotactic protein (MCP-1; CCL2), granulocyte-macrophage colony stimulating factor (GM-CSF), Eotaxin (CCL11), and tumor necrosis factor alpha (TNF-α). The Luminex™ system was used in accordance to manufacturer’s instructions. The median fluorescent value of each analyte was reported and compared with the fluorescent value of a standard curve with a range of 3.2 pg/mL to10,000 pg/mL for all analytes.

### Genotyping

The *Δ2bp* variant was genotyped using a custom (5’exonuclease, “TaqMan^®^”) allele discrimination assay in ABI7000 Sequence Detection System (SDS) and SDS software version 1.2.3 (Thermo Fisher Scientific, Waltham, MA, USA). The forward PCR primer was 5’-CGCTGGTTTCCCTTTGATGTG-3’; the reverse PCR primer was 5’-GGACCAGCCTCCGTAAGAC-3’; the reporter probe for the non-deletion was 5’-VIC-CTGCAAACTGAAGTTT-3’; and the reporter probe for the deletion allele was 5’-FAM-CACTGCAAACAAGTTT-3’. Cycling conditions were 95°C for 10 minutes followed by 50 cycles of 95°C for 15 seconds and 58° C for 1.5 minutes. Genotypes were called by two different individuals blinded to phenotypes with discrepancies decided by review of raw data or re-genotyping.

### Statistical analysis

All the statistic procedures were conducted in SPSS version 26. The analysis of interest was performed on each of the imputed datasets and resulting parameter estimates are combined using Rubin’s rules [27]. Independent sample t test was used to investigate the overall genetic effect, and univariate analysis in general linear model was used to evaluate the genetic effects on temporal effect of inflammatory mediators as well as on the association of inflammatory mediators and outcome measures. Alpha value is set at P = 0.05. The original and imputed data are available in S1 File (severe SCI) and S2 File (mild SCI).

### Outcome measures

Pain scale (range from 0 to 10) and Braden scale (range from 9 to 23) [28] were collected during each blood draw. The Numerical Pain Scale was used to measure sensitivity to neuropathic pain (lower score indicated lower pain) [29, 30]. The Braden scale is used to measure risk of pressure ulcers (lower score indicated higher risk).

### Missing data imputation

Multiple imputation on inflammatory mediators was conducted with SPSS version 26. We addressed the problem of missing data using the fully conditional specification (FCS) method with iterative Markov Chain Monte Carlo simulation. For each iteration and for each variable in the order specified in the variable list, the FCS method fits a single dependent variable using all other available variables in the model as predictors, then imputes missing values for the variable being fit. Predictive mean matching (PMM) model is used to scale variables. The method continues until convergence is reached, and the imputed values are saved to the imputed dataset. Little’s MCAR test with Expectation Maximation iteration till converge was used to test Missing Completely At Random (MCAR).

## Results

### Demographics

A total of 45 patients were included in this study, and 27 of the 45 patients were the *Δ2bp* carriers (Table 1). The *Δ2bp* carriers are defined as subjects with at least one copy of the *Δ2bp* variant. The *Δ2bp* non-carriers are defined as subjects without the *Δ2bp* variant. The severity of SCI was grouped according to the American Spinal Injury Association (ASIA) Impairment Scale (AIS). AIS grade A and B injuries were grouped as severe SCI (25 patients) and AIS grade C and D injuries were grouped as mild SCI (20 patients). In this dataset, 84% of the patients were male. The average age is 35.74 years in severe SCI group and 47.62 years in mild SCI group. Most of the injuries were cervical injuries and thoracic injuries. The causes of injuries were also listed in Table 1 for motor vehicular accident (MVA), gunshot wound (GSW), and fall. No significant difference on injury severity score (ISS) between Δ2bp carrier and Δ2bp non-carrier in severe (p = 0.09) and mild (p = 0.42) SCI. Higher ISS in severe SCI than in mild SCI was observed (p = 0.001). Acute days was not significant different between Δ2bp carrier and Δ2bp non-carrier in severe SCI (p = 0.63) and mild SCI (p = 0.26), but longer acute days in severe SCI than mild SCI was observed (p = 0.001). Most patients in this study were European Americans. The infections reported here were incidence of pneumonia, urinary tract infection (UTI), and pressure ulcer (PU). In severe SCI, infections between Δ2bp carrier and Δ2bp non-carrier were not significant in UTI (p = 0.99), pneumonia (p = 0.74), and PU (p = 0.99). In mild SCI, infections between Δ2bp carrier and Δ2bp non-carrier were not significant in UTI (p = 0.07), pneumonia (p = 0.76), and PU (p = 0.80). Significantly higher incidence of pneumonia and pressure ulcer were observed in severe SCI than in mild SCI (p = 0.001 and p = 0.0006, respectively), but not observed in UTI (p = 0.36). The raw data are provided in S1 File (for Severe SCI) and S2 File (for Mild SCI).

**Table 1:** Demographics of the SCI study population and *Δ2bp* genotype status of subjects for the *CHRFAM7AΔ2bp* variant. SD: standard deviation.

		Severe SCI	Mild SCI
Gender	Male	21	17
	Female	4	3
Age (Year±SD)		35.74±15.78	47.62±13.95
ASIA impairment scale		A(20), B(5)	C(10), D(10)
Injury Level		Cervical (10) Thoracic (15)	Cervical (13) Thoracic (3) Lumbar (4)
Injury cause		MVA (11)	MVA (2)
		GSW (5)	GSW (3)
		Fall (8)	Fall (10)
		Other (1)	Other (5)
ISS (Mean±SD)	Δ2bp carrier	33.3±10.5	16.7±7.8
	Δ2bp non-carrier	25.9±10.3	13.8±7.7
Acute Days (Mean±SD)	Δ2bp carrier	14±7	8±3
	Δ2bp non-carrier	16±12	7±3
Race	European American	*Δ2bp* (–) (7) *Δ2bp* (+) (13)	*Δ2bp* (–) (7) *Δ2bp* (+) (11)
	African American	*Δ2bp* (–) (3) *Δ2bp* (+) (2)	*Δ2bp* (–) (1) *Δ2bp* (+) (1)
UTI	Δ2bp carrier	*3*	*0*
	Δ2bp non-carrier	*2*	*2*
pneumonia	Δ2bp carrier	*8*	*1*
	Δ2bp non-carrier	*6*	*1*
PU	Δ2bp carrier	*9*	*2*
	Δ2bp non-carrier	*6*	*1*
Δ2bp	carrier	15	12
	non-carrier	10	8

GSW: Gunshot wound; MVA: Motor Vehicular accident. ISS: Injury severity score. UTI: incidence of Urinary trac infection. PU: incidence of Pressure Ulcer. SD: standard deviation. Pearson Chi-Square tests was used to compare infection incidences between genotypes as well as between Severe and Mild SCI groups.

### Missing data

Data were split into severe SCI and mild SCI groups. For the severe SCI injury group, outcome and biochemical data were available within six weeks of injury, and for the mild SCI group, data were from within three weeks post injury. Shorter hospitalization days were observed in mild SCI group and therefore three weeks of data points were included in mild SCI group. The missing rate of each of the variables is shown in Table 2. Different rates of missing were found among the inflammatory mediators in these two groups, ranging from 0 ~ 77% (Table 2). Variables with over 20% missing rate were excluded from further analysis. In the severe SCI group, four variables had > 20% missing rate. In mild SCI group, nine variables had > 20% missing rate. The dispersion of data were reported in relative standard error (RSE) [31]. Lower RSE represents lower dispersion of data. Multiple imputation was conducted for the randomly missing measurements of inflammatory mediators. Little’s MCAR tests were not significant for severe SCI (χ^2^ = 2258.08, degrees of freedom = 2320, P = 0.818) and the mild SCI group (χ^2^ = 1100.98, Degrees of freedom = 1057, P = 0.169). Thus, missing data can be considered random for the set of data and thus the cause of missing is unrelated to the data itself. MCMC/PMM multiple imputation was conducted and generated 20 sets of data for each group. Analyses run on each dataset were pooled according to Rubin’s rules and the reported results are pooled parameters of multiple imputation. The imputed data are provided in S2 File (for Severe SCI) and S3 File (for Mild SCI). The imputed data are provided in S3 File (for Severe SCI) and S4 File (for Mild SCI).

**Table 2:** Rate of missing for each inflammatory mediators and outcome measurements.

	Severe SCI		Mild SCI		
Variables	Missing (%)	RSE (%)	Variables	Missing (%)	RSE (%)
TNF-α	2.24	5.78	TNF-α	6.58	15.64
CCL11	0.45	4.36	CCL11	2.63	8.30
GM-CSF*	22.87	22.92	GM-CSF*	34.21	18.30
IFN-a2	16.59	7.23	IFN-a2*	28.95	13.17
IFN-γ	15.70	13.91	IFN-γ	1.53	16.78
IL-1ra	1.79	1.11	IL-1ra	9.21	16.35
IL-1b	19.28	16.93	IL-1b	3.36	19.48
IL-2	2.63	13.13	IL-2*	34.21	18.33
sIL-2Ra	17.49	6.16	sIL-2Ra*	27.63	19.83
IL-4*	65.02	16.32	IL-4*	77.63	34.34
IL-5	9.42	9.19	IL-5	2.63	9.43
IL-6	4.04	9.42	IL-6	5.26	55.72
IL-7*	47.09	8.59	IL-7*	39.47	18.64
IL-8	1.79	6.26	IL-8	3.95	16.04
IL-10	1.79	8.57	IL-10	17.11	15.70
IL-12p40	19.28	1.53	IL-12p40*	31.58	17.31
IL-12p70	6.73	19.22	IL-12p70	6.58	15.03
IL-13	15.25	7.20	IL-13*	38.16	19.65
IL-15	6.73	6.04	IL-15	3.95	1.66
IL-17*	24.66	11.87	IL-17*	39.47	2.75
CXCL10	0	6.96	CXCL10	0	7.93
CCL2	0	3.89	CCL2	0	6.63
CCL3	3.94	1.82	CCL3*	32.89	14.02
CCL4	5.38	7.74	CCL4	5.26	8.11
CXCL9	0	8.81	CXCL9	6.58	15.30
Painscale	2.18	5.76	Painscale	7.89	5.71
Bradenscale	1.31	3.24	Bradenscale	15.79	2.15

*: Variables with over 20% of missing rate were excluded from the analysis. RSE: Relative Standard Error.

### Overall genetic effect of *CHRFAM7AΔ2bp* variant on inflammatory mediators

Overall genetic effects were conducted by independent t-test on each inflammatory mediator to evaluate mean differences between the *Δ2bp* carriers and the non-carriers in each injury group. The data in Table 3 show the overall effect of *Δ2bp* variant on plasma levels of inflammatory mediators following SCI. The reported results were pooled results from multiple imputations according to Rubin’s rule.

**Table 3:** Overall genetic effect of *CHRFAM7AΔ2bp* variant.

	Severe SCI				Mild SCI			
	*Δ2bp* (–)	*Δ2bp* (+)	P	FDR	*Δ2bp* (–)	*Δ2bp* (+)	P	FDR
TNF-α	4.31±0.38	6.79±0.47	5x10^-5^	9.6x10^-4^	8.02±1.75	3.82±0.50	0.021	0.056
IFN-γ	8.64±1.75	27.81±3.84	1x10^-4^	1.3x10^-3^	15.56±3.64	7.32±1.13	0.031	0.062
IL-13	3.84±0.50	6.46±0.51	2x10^-4^	1.6x10^-3^				
CCL11	36.01±1.85	48.12±2.89	4x10^-4^	2.1x10^-3^	63.02±8.32	51.20±5.04	0.206	0.299
IL-12p70	4.85±1.52	21.18±4.48	5x10^-4^	2.2x10^-3^	8.70±1.91	5.17±0.84	0.092	0.163
IL-8	13.48±1.19	20.36±1.64	7x10^-4^	2.2x10^-3^	23.29±4.87	8.80±1.31	0.004	0.064
CXCL10	595.83±48.78	919.48±86.18	0.001	3.1x10^-3^	512.21±56.92	444.38±50.38	0.372	0.496
CCL4	23.67±1.87	36.39±3.78	0.003	5.7x10^-3^	36.80±4.39	25.64±4.24	0.027	0.062
IL-12p40	47.61±4.89	83.06±10.85	0.003	7.1x10^-3^				
IL-1b	5.42±0.69	12.05±2.38	0.007	0.014				
IL-15	9.26±0.90	12.47±0.92	0.013	0.024	11.54±1.76	6.28±0.69	0.005	0.040
IL-2	3.84±0.55	6.78±1.03	0.022	0.037				
IFN-a2	27.33±3.68	36.08±3.26	0.038	0.054				
IL-5	2.12±0.23	2.98±0.36	0.044	0.059	2.27±0.34	2.03±0.23	0.568	0.606
CCL2	319.22±18.60	375.82±19.27	0.039	0.060	291.07±19.86	217.46±23.72	0.019	0.061
IL-1ra	46.31±6.61	67.33±8.92	0.074	0.093	58.06±12.33	26.55±4.90	0.018	0.072
CXCL9	492.73±59.43	666.99±76.46	0.097	0.114	809.12±196.99	1054.34±223.86	0.395	0486
IL-10	64.92±6.96	74.85±9.15	0.40	0.407	23.35±4.75	19.39±4.31	0.534	0.610
sIL-2Ra	103.53±9.44	114.62±8.95	0.39	0.440				
IL-6	25.72±4.40	24.13±2.31	0.735	0.735	21.99±4.17	153.82±93.78	0.160	0.256
Braden Scale	13.32±0.24	15.01±0.85	0.088	0.114	15.83±0.48	16.21±0.49	0.592	0.592
Pain Scale	4.12±0.39	4.44±0.85	0.501	0.525	5.01±0.53	6.61±0.40	0.015	0.080

P: P-value. FDR: False discovery rate. *Δ2bp* (–) refers to the *Δ2bp* non-carriers. *Δ2bp* (+) refers to the *Δ2bp* carriers.

In the severe SCI group, the *Δ2bp* carriers had significantly higher levels of inflammatory mediators than the *Δ2bp* non-carriers, including TNF-α (FDR = 9.6x10^-4^), IFN-γ (FDR = 1.3x10^-3^), IL-13 (FDR = 1.6x10^-3^), CCL11 (FDR = 2.1x10^-3^), IL-12p70 (FDR = 2.2x10^-3^), IL-8 (FDR = 2.2x10^-3^), CXCL10 (FDR = 3.1x10^-3^), CCL4(FDR = 5.7x10^-3^), IL-12p40 (FDR = 7.1x10^-3^), IL-1b (FDR = 0.014), IL-15 (FDR = 0.024), and IL-2 (FDR = 0.037).

In the mild SCI group, the *Δ2bp* carriers have significantly lower levels of inflammatory mediators than the *Δ2bp* non-carriers for IL-15 (FDR = 0.04). Trends for lower plasma levels of TNF-α (FDR = 0.056), IFN-γ (FDR = 0.062), IL-8 (FDR = 0.064), CCL4 (FDR = 0.062), CCL2 (FDR = 0.061) and IL-1ra (FDR = 0.072) in the *Δ2bp* carriers than the *Δ2bp* non-carriers were also observed in mild SCI. In mild SCI, the pain scale is higher in the *Δ2bp* carriers than the *Δ2bp* non-carriers (P = 0.015), but not significant after FDR multiple comparison correction (Table 1). We have also stratified patients by their injury level above T6 or below T6 level (S1 Table). For injury level below T6, which generally considered higher impact on patients’ daily life after SCI than injuries above T6 level, the *Δ2bp* carriers also showed significantly higher levels of several inflammatory mediators than the *Δ2bp* non-carriers.

### Temporal variation of circulating inflammatory mediators and *CHRFAM7AΔ2bp* variant

We applied univariant analysis under a general linear model to investigate the genetic effect of the *Δ2bp* variant on the temporal variation of the inflammatory mediators post SCI. Again, the data used for analysis of the severe SCI group were within six weeks of injury, and the data used in mild SCI group were within three weeks of injury. The results in Table 4 show the pooled parameters estimates for the *Δ2bp* effect on temporal variation of inflammatory mediators. We evaluated the slopes of the temporal variation of the inflammatory mediators to see if the levels of these proteins were significantly different between the *Δ2bp* carriers and the non-carriers, and to further check if the slopes were significantly different from zero. The p-values reported here were pooled p-values of the 20 imputed data sets according to Rubin’s rule.

**Table 4:** *Δ2bp* genetic effects on temporal effect of inflammatory mediators.

pooled	Severe SCI				Mild SCI			
	B	Std.err	*T*	P	B	Std.err	*T*	P
TNF-α	0.027	0.058	0.469	0.639	-0.127	0.302	-0.421	.0674
CCL11	0.180	0.335	0.536	0.592	1.396	1.488	0.938	0.348
IFN-a2	0.188	0.399	0.470	0.638				
IFN-γ	-0.104	0.412	-0.253	0.801	0.250	0.509	0.490	0.624
IL-1ra	0.881	1.139	0.773	0.440	-0.410	1.856	-0.221	0.825
IL-1b	0.112	0.280	0.399	0.690				
IL-2	0.217	0.117	1.859	0.063				
sIL-2Ra	1.540	1.136	1.357	0.175				
IL-5	0.094	0.034	2.287	**0.020**	-0.046	0.071	-0.650	0.516
IL-6	-0.060	0.471	-0.128	0.898	-0.692	8.678	-0.080	0.936
IL-8	0.470	0.168	2.798	**5x10**^**-3**^	0.253	1.352	0.187	0.851
IL-10	0.433	0.886	0.488	0.625	0.468	0.822	0.569	0.569
IL-12p40	1.540	1.152	1.344	0.179				
IL-12p70	-0.212	0.534	-0.397	0.691	-0.366	0.366	-1.001	0.317
IL-13	0.101	0.066	1.538	0.124				
IL-15	-0.047	0.109	-0.435	0.664	0.000	0.242	0.002	0.999
CXCL10	-1.61	6.787	-0.238	0.812	-11.09	12.496	-0.887	0.375
CCL2	5.37	2.356	2.278	**0.023**	2.329	5.038	0.462	0.644
CCL4	0.367	0.428	0.858	0.391	-0.135	0.936	-0.144	0.885
CXCL9	-7.00	8.173	-0.856	0.392	26.156	50.798	0.515	0.607
painscale	-0.071	0.043	-1.66	0.098	0.032	0.138	0.233	0.816
bradenscore	0.037	0.074	0.505	0.614	0.088	0.203	0.434	0.664

B: Estimated parameter for the interaction term. std.err: standard error of B. T: t-test statistic. P: p-value

**Fig 1:**
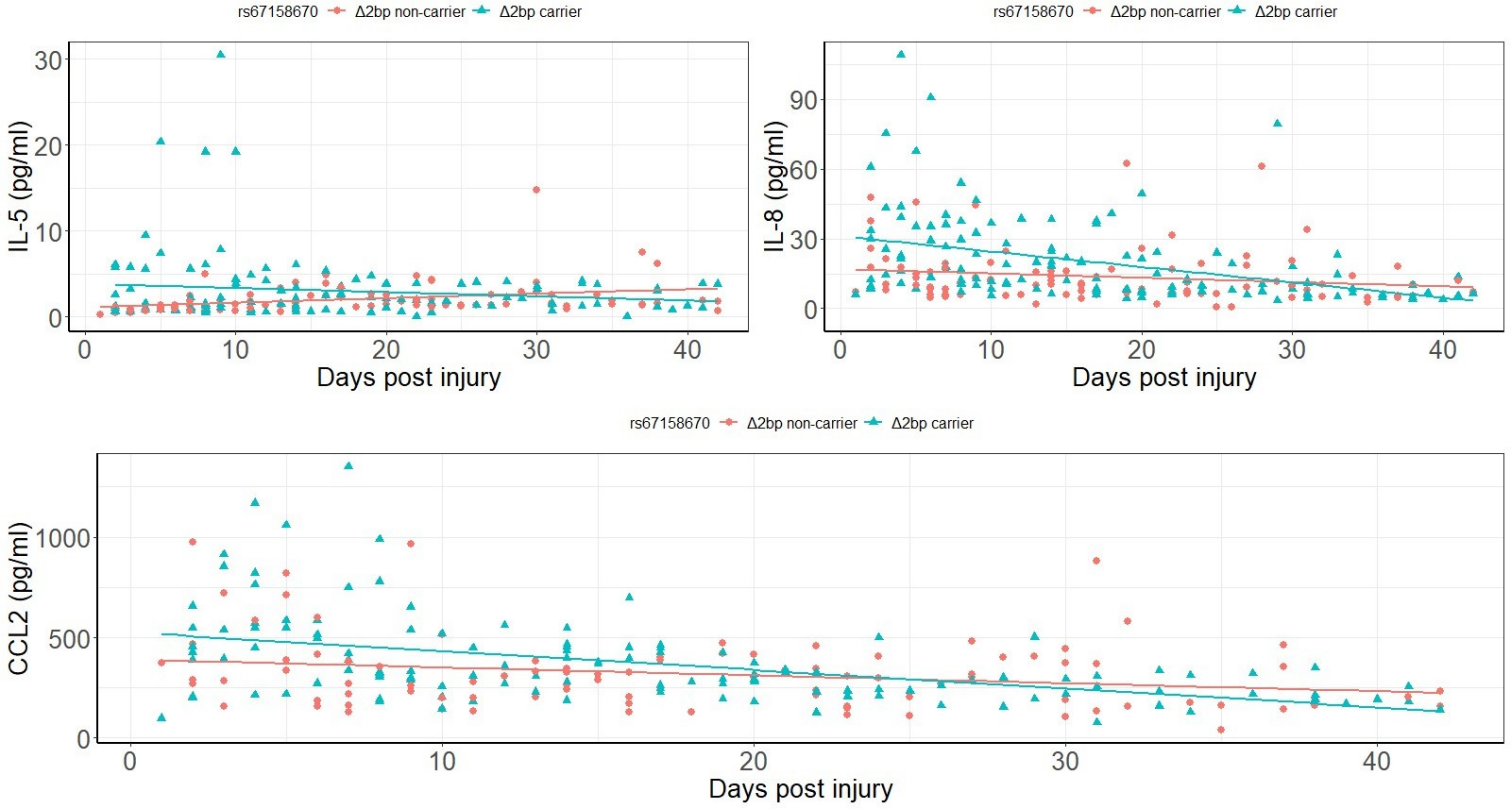
Temporal effect of inflammatory mediators in severe SCI is a function of genotype.

In severe SCI, univariate analysis showed that circulating levels of IL-5, IL-8 and CCL2 were a function of days post injury (DPI) and *Δ2bp* variant. The temporal variation of circulating levels of IL-5, IL-8 and CCL2 post SCI were significantly different between the *Δ2bp* carriers and the non-carriers (P = 0.02, 0.005 and 0.02, respectively). Further regression analysis revealed that IL-8 (adjusted R^2^ = 0.147, P = 2x10^-7^) and CCL2 (adjusted R^2^ = 0.217, P = 2x10^-8^) were negatively associated with DPI for the *Δ2bp* carriers, but the associations were not significant for the *Δ2bp* non-carriers (Fig 1). By contrast, the *Δ2bp* non-carriers showed positively associated IL-5 and DPI (adjusted R^2^ = 0.08, P = 0.015), but the association was not significant for the *Δ2bp* carriers (Fig 1).

In mild SCI, no significant genetic effect of *Δ2bp* variant on temporal effect of inflammatory mediators was observed.

### Genetic effect on the association of inflammatory mediators and outcome measures

In this section, we investigated the association of inflammatory mediators and outcome measures as a function of *Δ2bp* variant. The outcome measures used were numerical pain scale scores (NPS) and pressure ulcers risk as determined by the Braden scale [28]. We evaluated if the association slopes of outcome measures and inflammatory mediators were significantly different between the *Δ2bp* carriers and the *Δ2bp* non-carriers, and further check if the slopes were significantly different from zero. The p-values reported here are pooled p-values of the 20 imputed data sets according to Rubin’s rule. Higher pain scale indicated a greater degree of neuropathic pain. Lower Braden scale scores indicated a greater risk of pressure ulcers.

**Fig 2:**
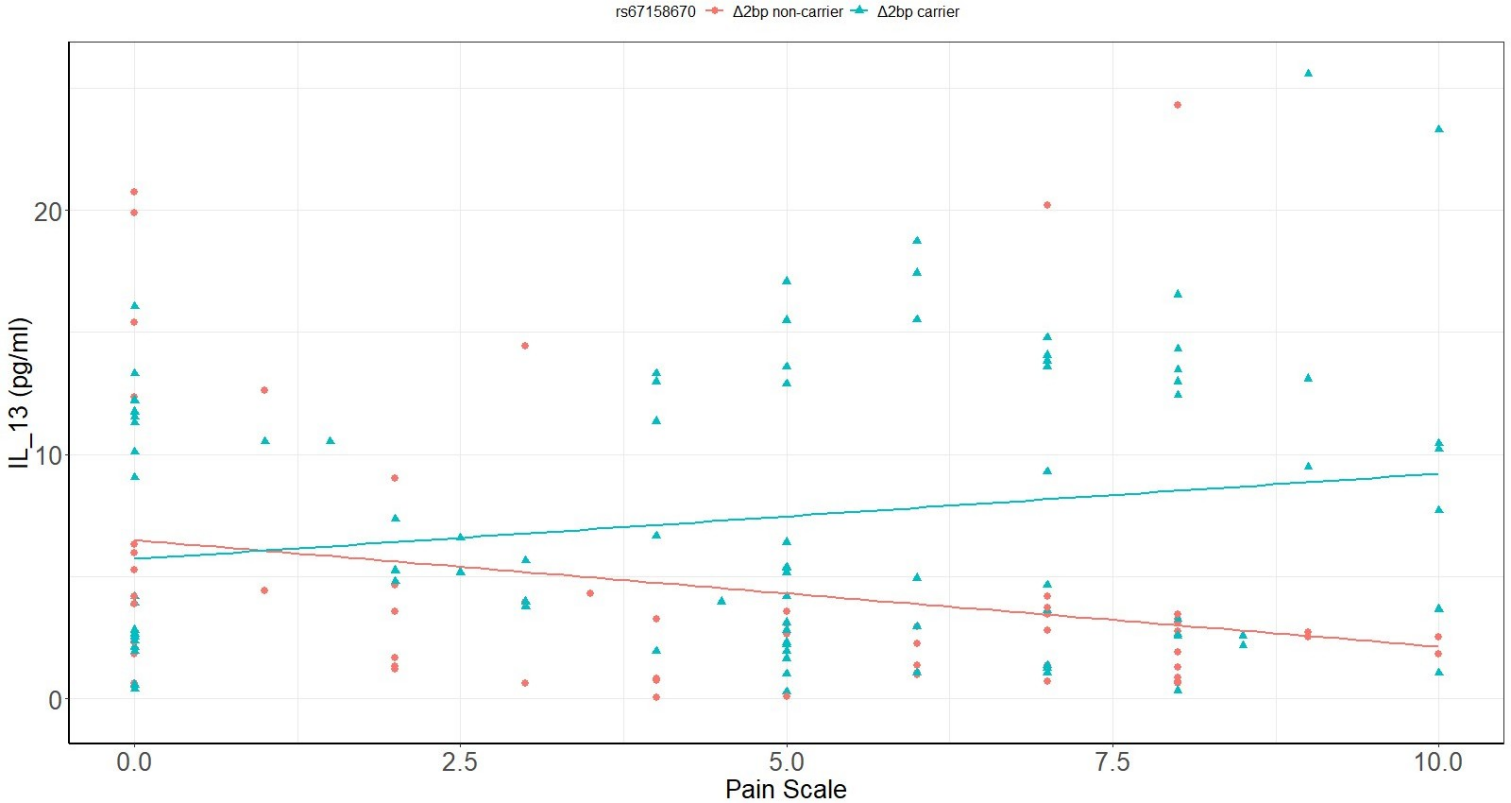
Association of IL-13 with pain scale in severe SCI is a function of *Δ2bp* variant.

In severe SCI, IL-13 is a function of pain scale and *Δ2bp* variant. The association of IL-13 with pain scale is significantly different between *Δ2bp* carriers and non-carriers (P = 0.013, Table 5, Fig 2). Further regression analysis showed that pain scale was marginally positively associated with IL-13 for *Δ2bp* carriers (adjusted R^2^ = 0.027, P = 0.056, Fig 2). For *Δ2bp* non-carriers, a trend of negatively associated pain scale with IL-13 was observed (P = 0.098, Fig 2). In mild SCI group, pain scale was not associated with inflammatory mediators and the *Δ2bp* variant.

**Table 5:** Genetic effects on association of inflammatory mediators and pain scale.

	Severe SCI				Mild SCI			
	B	Std.err	*T*	P	B	Std.err	*T*	P
TNF-α	0.116	0.127	0.914	0.361	0.064	0.189	0.341	0.733
CCL11	-0.009	0.010	-0.900	0.368	-0.012	0.017	-0.689	0.491
IFN-a2	-0.024	0.026	-0.917	0.359				
IFN-γ	-0.038	0.040	-0.957	0.340	-0.006	0.093	-0.059	0.953
IL-1ra	-0.011	0.017	-0.627	0.530	0.008	0.026	0.290	0.772
IL-1b	-0.007	0.070	-0.101	0.920	0.068	0.432	0.158	0.874
IL-2	-0.047	0.120	-0.394	0.693				
sIL-2Ra	-0.003	0.005	-0.550	0.583				
IL-5	0.050	0.260	0.190	0.849	0.068	0.432	0.158	0.874
IL-6	0.006	0.016	0.365	0.716	-0.025	0.017	-1.438	0.151
IL-8	0.033	0.038	0.861	0.390	-0.018	0.040	-0.442	0.658
IL-10	0.004	0.003	1.367	0.172	0.043	0.026	1.663	0.096
IL-12p40	-0.009	0.010	-0.949	0.343				
IL-12p70	0.000	0.120	0.001	0.999	0.043	0.113	0.380	0.704
IL-13	-0.23	0.092	-2.499	**0.013**				
IL-15	-0.029	0.045	-0.632	0.527	-0.018	0.119	-0.150	0.881
CXCL10	0.000	0.001	-0.308	0.758	0.001	0.003	0.516	0.606
CCL2	0.003	0.003	1.144	0.253	-0.002	0.007	-0.342	0.733
CCL4	-0.006	0.031	-0.197	0.844	-0.032	0.035	-0.910	0.363
CXCL9	0.000	0.001	-0.290	0.772	-0.001	0.001	-1.120	0.263

B: Estimated parameter for the interaction term. std.err: standard error of B. T: t-test statistic. P: p-value

**Fig 3:**
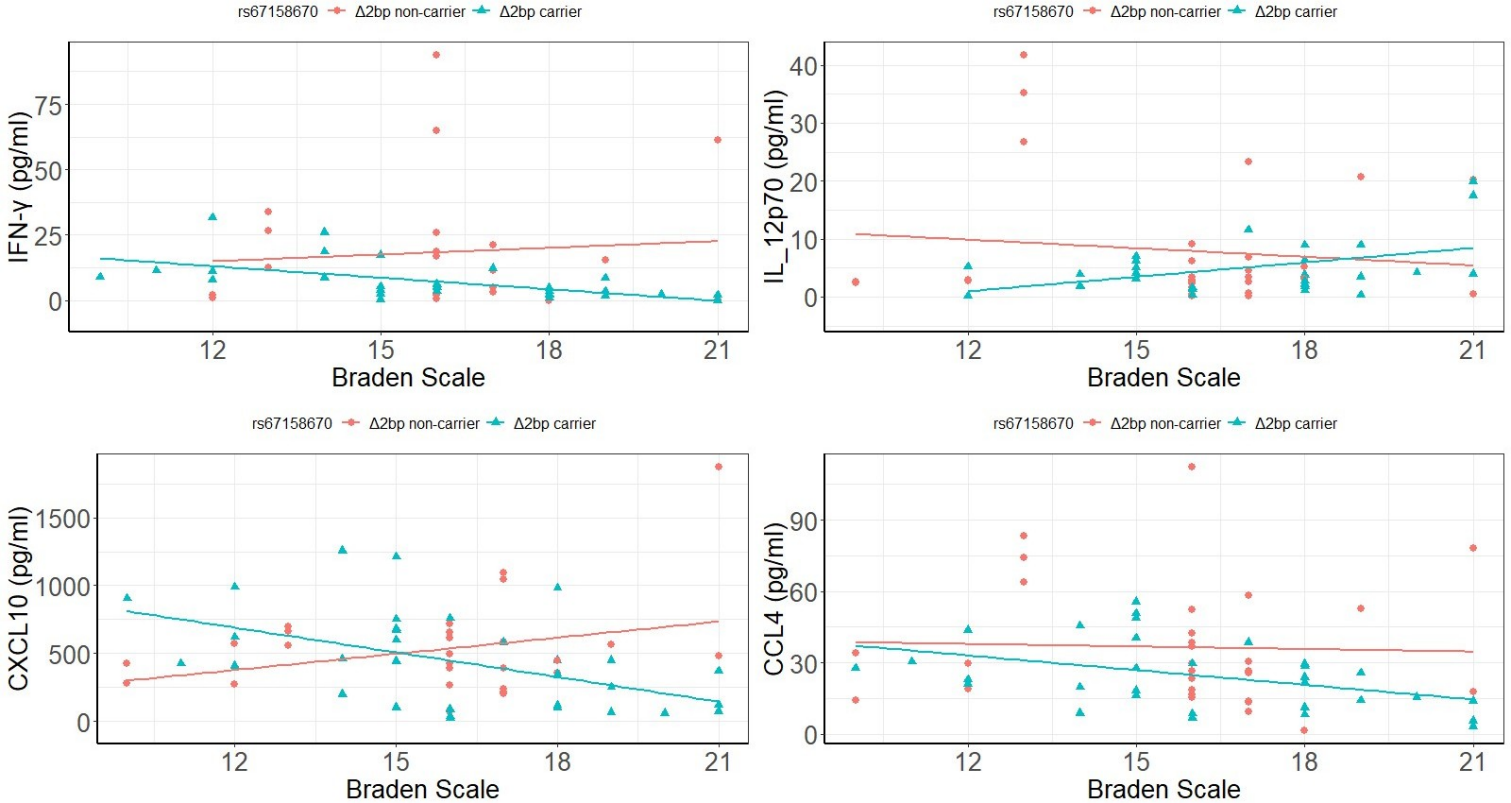
Association of inflammatory mediators with Braden scale in mild SCI is a function of genotype.

In mild SCI, the interactions of IFN-γ (P = 1.4x10^-4^), IL-12p70(P = 8x10^-3^), CXCL10 (P = 5x10^-3^), and CCL4 (P = 0.02) with Braden scale were significantly associated with *Δ2bp* variant (Table 6, Fig 3). Further regression analysis revealed that Braden scale were negatively associated with circulating levels of IFN-γ (adjusted R^2^ = 0.304, P = 9x10^-5^), CXCL10 (adjusted R^2^ = 0.221, P = 0.003) and CCL4 (adjusted R^2^ = 0.162, P = 0.007) in the *Δ2bp* carriers, but not in *Δ2bp* non-carriers (Fig 3). Braden scale was positively associated with circulating levels of IL-12p70 (adjusted R^2^ = 0.135, P = 0.021) in *Δ2bp* carriers but not in *Δ2bp* non-carriers (Fig 3). Thus, for the *Δ2bp* carriers in mild SCI, a higher risk of pressure ulcers was associated with higher circulating levels of IFN-γ, CXCL10, and CCL4 and lower circulating level of IL-12p70.

**Table 6:** Genetic effects on association of inflammatory mediators and braden scale.

	Severe SCI				Mild SCI			
	B	Std.err	*T*	P	B	Std.err	*T*	P
TNF-α	0.314	0.173	1.817	*0*.*069*	0.120	0.099	1.213	0.225
CCL11	0.013	0.022	0.582	0.561	0.022	0.013	1.672	0.095
IFN-a2	-0.008	0.016	-0.527	0.599				
IFN-γ	0.017	0.023	0.706	0.480	0.237	0.062	3.808	**1.4x10**^**-4**^
IL-1ra	0.006	0.008	0.817	0.414	0.015	0.019	0.751	0.453
IL-1b	0.045	0.034	1.343	0.180				
IL-2	0.000	0.166	-0.003	0.998				
sIL-2Ra	-0.001	0.007	-0.138	0.890				
IL-5	0.244	0.179	1.363	0.173	-0.804	0.487	-1.651	0.104
IL-6	-0.009	0.027	-0.337	0.736	-0.034	0.023	-1.453	0.146
IL-8	-0.001	0.039	-0.027	0.978	0.092	0.096	0.956	0.339
IL-10	0.006	0.012	0.535	0.592	0.034	0.028	1.210	0.226
IL-12p40	0.000	0.006	0.049	0.961				
IL-12p70	0.017	0.053	0.314	0.754	-0.283	0.106	-2.669	**8x10**^**-3**^
IL-13	0.135	0.074	1.831	*0*.*067*	0.070	0.175	0.401	0.689
IL-15	-0.124	0.125	-0.992	0.321	0.151	0.128	1.180	0.238
CXCL10	0.000	0.001	0.324	0.746	0.006	0.002	2.831	**5x10**^**-3**^
CCL2	0.003	0.003	0.948	0.345	0.003	0.004	0.789	0.430
CCL4	0.007	0.016	0.441	0.659	0.083	0.034	2.432	**0.015**
CXCL9	0.002	0.001	1.387	0.166	0.000	0.001	0.175	0.861

B: Estimated parameter for the interaction term. std.err: standard error of B.

T: t-test statistic. P: p-value

In severe SCI, the associations of Braden scale and circulating levels of inflammatory mediators as a function of the *Δ2bp* variant were not observed.

## Discussion

Segmental duplications of genes in recent human evolution and adaptation introduced large-scale structural variation in their encoded proteins and produced new gene functions. These human-specific genes are important factors in complex human diseases [32] and the *CHRFAM7A* gene is an example of one of these genes. In this study, we observed that genetic impact of *Δ2bp* variant on inflammation is more evident in individuals with severe SCI than those with mild SCI, and that the *Δ2bp* carriers had higher circulating levels of several inflammatory mediators than the *Δ2bp* non-carriers in severe SCI (Table 3). In severe SCI, temporal variation of IL-5, IL-8, and CCL2 levels following SCI were associated with the *Δ2bp* variant, and NP was a function of circulating IL-13 level and the *Δ2bp* variant. In mild SCI, the risk of pressure ulcers was associated with circulating levels of IFN-γ, IL-12p70, CXCL10, and CCL4, and the associations were influenced by the *Δ2bp* variant. Together, the results of this study suggested that the *Δ2bp* variant may influence circulating levels of inflammatory proteins following SCI that may also influence clinical outcomes based on injury severity. This idea is supported by the findings of other investigators whose goal was to stratify patients by injury severity in order to accurately predict clinical outcomes (reviewed by [33]). Earlier studies by Kwon et al [34] on levels of inflammatory cytokines in cerebral spinal fluid (CSF) and blood from acutely injured SCI subjects stratified by injury severity showed a rapid increase in CSF cytokines during the initial 24–48 hrs following injury. Among the cytokines measured, CSF levels IL-6, IL-8, and MCP-1 provided predictive information on injury severity and recovery from injury. CSF levels of these proteins were predictive of recovery from early days (3–4 days) to many weeks following injury. By comparison, serum cytokine levels in the same set of SCI subjects, where as much as three orders of magnitude lower than CSF levels [34]. Therefore, the authors suggested that CSF may provide the best source of biofluid to determine patterns of cytokine levels in subjects with SCI subjects. Because there are physical and clinical issues in obtaining CSF samples in acutely SCI patients, we relied on plasma as a biofluid. We obtained biosamples in the days and weeks following injury for our studies. Despite differences in methodology between our group and Kwon et al, our findings also support a role for TNF and IL-8 as part of the cytokine profile produced by subjects with severe SCI. However, in contrast to Kwon et al (2010), who did not find an association of inflammatory cytokines with NP in SCI subjects stratified by injury severity (up to 25 DPI), we found differences in NP measured by NPS when severely injured SCI subjects were stratified by *CHRFAM7A Δ2bp* genotype.

The innate immune system and inflammatory responses have been identified as key causative components of secondary inflammation after SCI [35]. In the peripheral nervous system, recent studies suggested that the vagus nerve acts as an endogenous cholinergic anti-inflammatory pathway, and is composed of a regulatory arch composed of the efferent vagus nerve, the neurotransmitter acetylcholine (ACh), and the α7nAChRs [36]. The α7nAChRs act as molecular link between the brain and the innate immune system [37, 38]. Experiments with α7nAChRs knockout mice established that α7nAChRs were essential for vagal inhibition of the inflammatory response to Lipopolysaccharides (LPS) and caused a larger increase of serum TNF-α than wild-type controls [11]. The same study also demonstrated expression of α7nAChRs mRNA by human macrophages. Thus, ACh inhibited LPS-stimulated release of TNF-α from macrophages by stimulating α7nAChRs and activating a signaling mechanisms likely involving G proteins [13, 39]. Our results suggest that the *Δ2bp* carriers, which are predicted to express functionally compromised α7nAChRs due to absence of protein glycosylation and the lack of a cysteine bridge [20], exhibit higher levels of circulating pro-inflammatory cytokines in subjects with severe SCI. These findings support the importance of α7nAChRs in the cholinergic anti-inflammatory pathway and suggest that the endogenous *Δ2bp* variant acts as an inhibitor of the α7nAChR anti-inflammatory function. The differential impact on TNF-α by *Δ2bp* carriers and non-carriers in severe SCI also suggests critical role of α7nAChRs on circulating TNF-α levels. This is consistent with our previous findings on interactions between TNF-α, norepinephrine, and the *Δ2bp* variant on SCI inflammation and neuropathic pain [25].

In examining the temporal changes following severe SCI, an association of the *Δ2bp* variant on circulating levels of inflammatory mediators was observed. In severe SCI, *Δ2bp* carriers had increased plasma levels of CCL2 and IL-8 levels during the acute phase post SCI than the *Δ2bp* non-carriers. CCL2 is one of the key chemokines that regulates migration and infiltration of monocytes and macrophages [40] and is likely to play an important role in the early phase of post SCI inflammation, especially between twelve hours and two days after a CNS lesion [41, 42]. Notably, CCL2 is also a key biomarker for adverse outcomes following blunt force head trauma in humans [43]. Interleukin-8 is a key mediator associated with inflammation where it plays a key role in neutrophil recruitment and neutrophil degranulation [44] and traditionally been considered as detrimental and unfavorable to proper tissue regeneration, although recent data show that neutrophils could have an indirect beneficial effect for promoting functional recovery after spinal cord trauma [45]. Our results further support the idea that the *Δ2bp* variant increases circulating levels of pro-inflammatory cytokines in individuals with severe SCI and weakens the anti-inflammatory response of α7nAChRs in cholinergic pathways.

The fast response of α7nAChRs to inflammation is closely related to a permeability for calcium and affinity to ACh. Classic α7nAchRs are composed of five identical α7 subunits that form a central pore with high permeability for calcium [46]. With the introduction of a functionally compromised α7nAChRs subunits by the *Δ2bp* variant in humans, our data indicated significantly elevated circulating levels of several pro-inflammatory cytokines such as IL-12, TNF-α, and IFN-γ following severe SCI. In this study, circulating levels of IL-13 were also found higher for the *Δ2bp* carriers than the *Δ2bp* non-carriers in severe SCI.

To explore associations of pro- and anti-inflammatory cytokines with clinical outcomes in SCI, we incorporate measures of NP and the development of pressure ulcers into genotype-based stratified analysis. Our data from acute severe SCI human subjects indicated that *Δ2bp* non-carriers had a trend of negatively associated plasma levels of IL-13 and NP. In contrast, *Δ2bp* carriers had a marginally positive association of IL-13 with neuropathic pain. The findings with *Δ2bp* non-carriers are consistent with results from a mouse SCI model that showed that IL-13 was negatively correlated with NP [47]. In this context, a higher IL-13 level was associated with reduced NP. However, the impact of a compromised α7nAChR in *Δ2bp* carriers could produce the opposite result by failing to alleviate NP in humans. This underscores the importance of knowing *Δ2bp* carrier status as this could identify individuals with a fully functional (normal) α7nAChR who may benefit from selective α7nAChR ligands for pain control [48, 49] and differentiate them from non-responders. Additional experimentation and analysis is required to test this hypothesis.

The Braden Scale is used to assess risk for pressure ulcers and considers many factors including moisture, mobility, friction, and shear. Pressure injuries are a global problem affecting approximately 2.5 million patients per year. It can drastically impact the patients’ quality of life and increased costs to treat [50]. In this study, we only observed Braden scale as a function of circulating levels of inflammatory mediators and the *Δ2bp* variant in mild SCI. This could because that the items used in Braden scale such as sensory perception, mobility, and nutritional variables are may be undetectable and not significant variables in pressure ulcer estimate in individuals with severe SCI. For the *Δ2bp* carriers in mild SCI, our data suggested that these individuals were at higher risk of pressure ulcers and were associated with higher circulating levels of IFN-γ, CXCL10, and CCL4 but lower circulating levels of IL-12p70. Thus, a functionally compromised α7nAChR may be related to risk of pressure ulcers. CXCL10 is a CXC subfamily chemokine also known as IFN-γ Inducible Protein 10 (IP‐10), and has a role as a chemoattractant of several immune cells implicated in the pathology of several CNS disorders [51, 52]. Neutralization of CXCL10 reduced T-lymphocyte invasion and significantly enhanced tissue preservation and functional outcome in a mouse dorsal hemisection SCI model [53]. Neutralization of CXCL10 also reduced apoptosis [54] and enhanced tissue sparing, angiogenesis, and subsequent axon sprouting [55]. IL-12 stimulates the production of interferon-gamma (IFN-γ) and tumor necrosis factor-alpha (TNF-α) from T cells and natural killer (NK) cells, and reduces IL-4 mediated suppression of IFN-γ [56]. Therefore, functionally compromised α7nAChRs may adversely influence wound healing through the interaction of IL-12, IFN-γ, CXCL10 and CCL4, and an increased inflammatory response [57, 58]. Further studies are required to define the cell types and underlying mechanisms.

## Limitations

In addition to the aforementioned limitations associated with the fact that correlation does not equal causality, there are multiple other limitations of this study. SCI is a complex traumatic injury with several confounding factors. The inflammatory mediators measured in this study represent a subset of factors that can contribute to an individual’s response to the injury. Other factors may also influence the interaction of mediators investigated in this study. We strived to eliminate confounding factors in statistical analyses and approaches to find the most significant influence of the *CHRFAM7A* gene. However, with the limitation in sample size and the number of mediators investigated, our results should be interpreted with caution until a larger study population is recruited and the study replicated. Other genes may also have an impact on the cholinergic anti-inflammatory response and therefore, additional studies that involve more genes and outcome measures are necessary to explore the interaction of *CHRFAM7A* and other genes, and their association with inflammation and their contribution to SCI patient outcomes. Finally, it is noted that some results were computationally derived and should be interpreted with caution for clinical application.

## Supporting information

S1 Table The genetic effect of CHRFAM7A *Δ2bp* variant on inflammatory mediators stratified by injury above T6 and below T6. (DOCX)

S1 File Raw data for severe SCI. (XLSX)

S2 File Raw data for mild SCI. (XLSX)

S3 File Imputed data for severe SCI. (XLSX)

S4 File Imputed data for mild SCI. (XLSX)
