# Supplementary material for: Effect of CHRFAM7A Δ2bp gene variant on secondary inflammation after spinal cord injury
Source: PLoS One. 2021 May 6;16(5):e0251110. doi: 10.1371/journal.pone.0251110 (PMC8101719; doi:10.1371/journal.pone.0251110)
Supplement: S1 Table — (DOCX) [file pone.0251110.s001.docx]

**S1 Table.** Plasma levels of inflammatory mediators stratified by injury above T6 or below T6 level.

|  | Above T6 | | | Below T6 | | |
| --- | --- | --- | --- | --- | --- | --- |
|  | *∆2bp* (–) | *∆2bp* (+) | P | *∆2bp* (–) | *∆2bp* (+) | P |
| TNF-α | 4.39±0.41 | 6.41±0.62 | 0.007 | 7.69±1.67 | 5.54±0.61 | 0.08 |
| IL-12p70 | 4.97±1.54 | 14.05±4.18 | 0.04 | 8.25±1.85 | 21.76±5.75 | 0.03 |
| IL-1b | 6.48±0.88 | 12.85±3.22 | 0.06 | 3.94±0.57 | 6.69±0.80 | 0.005 |
| CCL2 | 337.88±18.15 | 346.81±23.58 | 0.77 | 241.23±19.19 | 321.47±21.80 | 0.006 |
| CXCL10 | 606.05±50.70 | 674.34±61.91 | 040 | 486.85±42.99 | 985.56±137.30 | 0.001 |
| CXCL9 | 603.16±86.03 | 605.70±86.77 | 0.98 | 500.33±101.92 | 991.60±154.29 | 0.008 |
| CCL11 | 35.17±3.20 | 38.85±3.20 | 0.36 | 45.99±3.54 | 55.81±3.75 | 0.06 |
| IFN-γ | 9.73±2.02 | 15.94±2.96 | 0.08 | 12.41±2.57 | 27.03±5.77 | 0.02 |
| IL-1ra | 49.76±7.19 | 67.94±11.26 | 0.18 | 48.36±9.07 | 41.02±4.18 | 0.45 |
| IL-6 | 24.04±4.17 | 76.96±39.69 | 0.19 | 26.64±5.90 | 26.51±3.68 | 0.98 |
| IL-8 | 16.91±2.07 | 17.78±1.89 | 0.76 | 13.72±1.69 | 17.08±1.78 | 0.22 |
| Il-10 | 51.34±6.16 | 45.31±7.23 | 0.53 | 61.39±11.95 | 83.92±13.79 | 0.29 |
| Il-15 | 10.00±0.98 | 10.15±0.99 | 0.91 | 9.47±1.39 | 12.07±1.10 | 0.14 |
| CCL4 | 27.43±2.13 | 35.85±4.72 | 0.10 | 26.22±3.87 | 30.65±2.11 | 0.32 |
| IL-13 | 4.50±0.68 | 5.26±0.55 | 0.39 | 3.54±0.61 | 7.18±0.75 | 0.002 |
| IL-12p40 | 57.97±7.54 | 90.20±15.33 | 0.06 | 55.59±10.04 | 61.18±6.40 | 0.63 |
| CCL4 | 27.43±2.13 | 35.85±4.72 | 0.10 | 26.22±3.87 | 30.65±2.11 | 0.32 |
| IFN-a2 | 24.13±2.45 | 35.69±4.22 | 0.02 | 50.55±7.19 | 32.51±3.38 | 0.024 |
| IL-2 | 5.20±0.88 | 7.47±1.59 | 0.21 | 4.12±0.81 | 5.79±0.61 | 0.10 |

For injuries below T6, the ∆2bp carriers had higher inflammatory mediator levels in plasma for IL-12p70, IL-1b, CCL2, CXCL10, CXCL9, IFN-γ, IL-13, and IFN-a2 than the ∆2bp non-carriers.

For injuries above T6, the ∆2bp carriers has higher inflammatory mediator levels in TNF-α , IL-12p70, and IFN-a2 than the ∆2bp non-carriers. We also observed higher overall TNF-α levels for injuries below T6 than above T6.

Thus, the anti-inflammatory function of CHRNA7 was aggravated by the ∆2bp allele more evidently for injuries below T6. And the lower lumbar injuries inhibited patients’ daily function more severely than the upper lumbar injuries, though both are quite serious injuries.
